# Confinement-deconfinement transition due to spontaneous symmetry breaking in quantum Hall bilayers

**DOI:** 10.1038/ncomms10462

**Published:** 2016-01-25

**Authors:** D. I. Pikulin, P. G. Silvestrov, T. Hyart

**Affiliations:** 1 Department of Physics and Astronomy, University of British Columbia, Vancouver, British Columbia, Canada V6T 1Z1; 2 Quantum Matter Institute, University of British Columbia, Vancouver, British Columbia, Canada V6T 1Z4; 3 Instituut-Lorentz, Universiteit Leiden, P.O. Box 9506, 2300 RA Leiden, The Netherlands; 4 Institute for Mathematical Physics, TU Braunschweig, 38106 Braunschweig, Germany; 5 Department of Physics and Nanoscience Center, P.O. Box 35 (YFL), FI-40014, University of Jyväskylä, Finland

## Abstract

Band-inverted electron-hole bilayers support quantum spin Hall insulator and exciton condensate phases. Interest in quantum spin Hall effect in these systems has recently put them in the spotlight. We investigate such a bilayer in an external magnetic field. We show that the interlayer correlations lead to formation of a helical quantum Hall exciton condensate state. Existence of the counterpropagating edge modes in this system results in formation of a ground state spin-texture not supporting gapless single-particle excitations. The charged edge excitations in a sufficiently narrow Hall bar are confined: a charge on one of the edges always gives rise to an opposite charge on the other edge. Magnetic field and gate voltages allow the control of a confinement-deconfinement transition of charged edge excitations, which can be probed with nonlocal conductance. Confinement-deconfinement transitions are of great interest, not least because of their possible significance in shedding light on the confinement problem of quarks.

## Abstract (web summary)

Electronic systems with inverted band structures can support exotic topological insulator and exciton condensate states. Here, the authors demonstrate the formation of a helical exciton condensate in quantum Hall bilayers, and a quark-like quasiparticle confinement-deconfinement transition.

## Graphical abstract

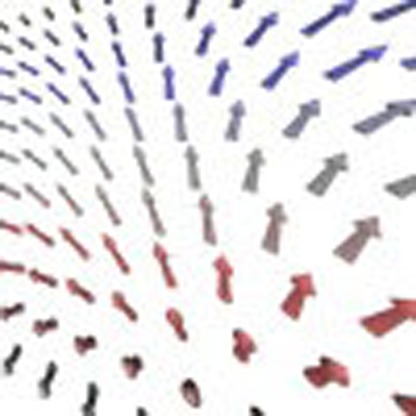

The role of the electron–electron interactions for the experimentally accessible topological media is best appreciated in quantum Hall (QH) systems. The fractionally charged quasiparticles have been studied at the fractional filling factors^1^,^2^, and the non-abelian excitations of more exotic QH states may eventually lead to a revolution in quantum computing^3^,^4^,^5^,^6^,^7^. However, Coulomb interactions play a crucial role also in the case of the integer filling factors^1^,^8^,^9^,^10^,^11^,^12^,^13^. Remarkably, interactions create a QH ferromagnetic ground state at total filling fraction *v*=1 even in the absence of Zeeman energy. In such systems, the *SU*(2) spin rotation symmetry is spontaneously broken, resulting in the low-energy excitations being spin waves and charged topological spin textures, skyrmions^1^,^8^,^12^,^13^. The presence of a small symmetry-breaking Zeeman field does not change the low-energy excitations qualitatively.

In QH bilayer systems the role of spin is played by the layer index (pseudospin)^8^,^10^,^11^,^13^. In this case the *SU*(2) pseudospin rotation symmetry is explicitly broken by the interactions, as they are larger within the layers than between the layers. The interactions favour the pseudospin orientations in (*x*, *y*)-plane, where the direction is chosen spontaneously (spontaneous *U*(1) symmetry breaking) so that the QH bilayers realize an easy-plane ferromagnet. Since the spontaneously chosen direction in the (*x*, *y*)-plane corresponds to a spontaneous interlayer phase coherence, this easy-plane ferromagnetic state is equivalent to an exciton condensate^11^,^13^.

The QH ferromagnet and QH exciton condensate in electron–electron bilayers support a single chiral edge mode. However, the two Landau levels may also support counterpropagating edge modes. The natural hosts of such kind of QH states are systems supporting quantum spin Hall (QSH) effect^14^,^15^,^16^,^17^,^18^,^19^ due to inverted electron-hole bandstructure. In these materials the magnetic field allows tuning through the Landau level crossing^20^,^21^, where we expect to find a QH state with spontaneously broken (pseudo)spin-rotation symmetry. Thus, we argue that there exist four different experimentally accessible pseudospin ferromagnetic states, determined by spontaneously broken symmetry (SU(2) in single layer and U(1) in bilayer systems) and the edge structure (chiral or helical). All these possibilities are illustrated in Fig. 1.

In this paper, we concentrate on the helical QH exciton condensate state (broken U(1) symmetry and helical edge structure). Remarkably, we find that in this system the charged edge excitations in a sufficiently narrow Hall bar are confined: a charge on one of the edges is always connected to the opposite charge on the other edge through the bulk by a stripe of rotated pseudospins, and thus low-energy isolated charged excitations cannot be observed. The gapless single-particle excitations are prohibited since the electron–electron interactions lead to an edge reconstruction and opening of a single-particle gap^22^,^23^. However, unlike it happens in the existing examples, the helical exciton condensate creates long-range correlations between edges. We show that a magnetic field and gate voltages can be used to tune in and out of the exciton condensate phase. Thus this system provides a unique opportunity to study a confinement-deconfinement transition, similar to the one which is hypothesized to liberate the quarks from their color confinement at extremely high temperatures or densities^24^. Finally, we show that the confined and deconfined phases can be distinguished using non-local conductance.

## Results

### Helical quantum Hall exciton condensate phase

**Figure 2:**
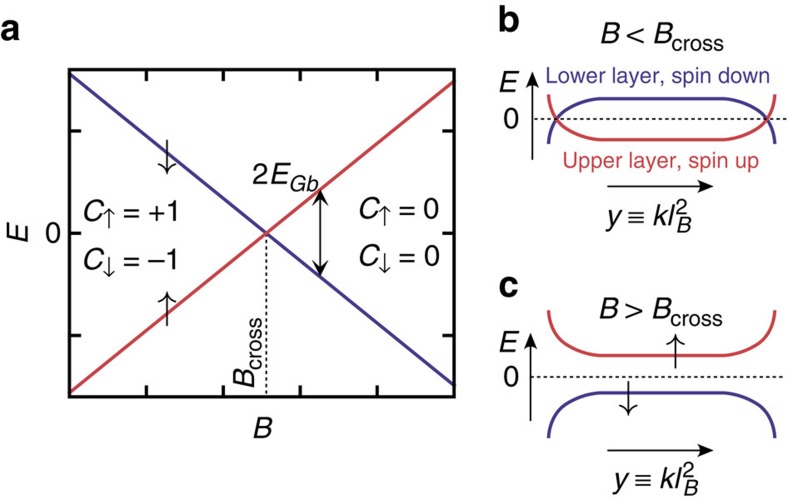
Magnetic field and momentum dependence of the lowest Landau level energies. (**a**) There exists a robust crossing of the lowest Landau level energies as a function of magnetic field at *B*=*B*_cross_, because the energy of the electron-like Landau level with spin up (red line) increases and the energy of the hole Landau level with spin down (blue line) decreases as a function of *B*. We denote the energy separation between these two levels as 2*E*_Gb_. (**b**,**c**) Momentum (or equivalently position) dependencies of the lowest Landau level energies. The electron-like (hole-like) Landau level bends upwards (downwards) in energy near the edge of the sample. (**b**) For *B*<*B*_cross_ the system supports helical edge modes protected by spin-resolved Chern numbers 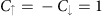. (**c**) For *B*>*B*_cross_ the edge is gapped according to the non-interacting theory (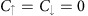).

We consider bilayer QSH systems, such as InAs/GaSb^17^,^18^,^19^, described by the BHZ Hamiltonian^15^,^17^ (Supplementary Note 1). The important property of these systems is that there is a crossing of electron and hole Landau levels as a function of magnetic field at *B*=*B*_cross_ (refs 20, 21; Fig. 2a and Supplementary Fig. 1), where the band inversion is removed. Near this crossing the single-particle Hamiltonian is (Supplementary Note 1):


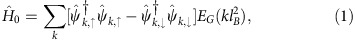


where 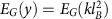 is the energy–momentum dispersion of Landau levels and 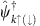 are the electron creation operators for the lowest electron and hole Landau levels. Here we have fixed the total filling factor of the Landau levels 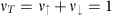, and used the fact that the momentum *k* in the Landau level wavefunctions is directly connected to the position *y* in the real space. Importantly, the spin and layer degrees of freedom are locked with each other, so that the pseudospin 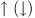 means simultaneously up (down) spin and upper (lower) layer. The Fermi level is set to be at zero energy.

In the bulk the energy *E*_*G*_(*y*)=*E*_Gb_ is independent of the momentum (*E*_Gb_<0 for *B*<*B*_cross_ and *E*_Gb_>0 for *B*>*B*_cross_). When approaching the edge the Landau level originating from the electron (hole) band always disperses upwards (downwards) in energy. The spatial variation of *E*_*G*_(*y*) occurs within a characteristic length scale 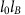, which depends on the details of the edge, but due to topological reasons *E*_*G*_(*y*)>0 reaches extremely large values (on the order of the energy separation between the bulk Landau levels) close to the edge (Supplementary Note 1). Therefore, for the magnetic fields *B*<*B*_cross_, *E*_*G*_(*y*) goes through zero near the edge, yielding to the helical edge states (Fig. 2b). On the other hand for *B*>*B*_cross_ the edge is gapped in the non-interacting theory (Fig. 2c).

The electron–electron interactions 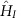 are described by


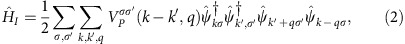


where 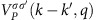 is obtained by projecting the Coulomb interactions to the subspace generated by the wavefunctions of the lowest Landau levels (Supplementary Note 2). Here we assumed that the higher Landau levels are energetically separated from the lowest ones by an energy gap larger than the characteristic energy scale of the Coulomb interactions 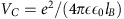. We find that this assumption can be satisfied with the material parameters corresponding to InAs/GaSb bilayers^25^.

To find the ground state of the Hamiltonian 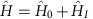, we consider states where the local direction of the pseudospin **h**(**r**) (|**h**(**r**)|=1) varies in space. Because the Hamiltonian is translationally invariant in the *x* direction, we assume that **h**(**r**) is independent of *x* (It is known that for sufficiently large interlayer separation the quantum Hall bilayers display an instability towards formation of a charge density wave ground state^26^. Here we assume that the interlayer separation is small enough that such kind of instability does not occur.). By further noticing that the *y*-dependence translates to a momentum dependence of the pseudospin 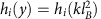, we can express our ansatz for the ground state many-particle wavefunction as a Slater determinant 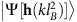, where for each momentum *k* we create an electron with pseudospin pointing along 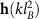. To compute the ground state, we need to minimize the energy functional for such kind of pseudospin texture^13^. For the energy functional we obtain (Supplementary Note 2)


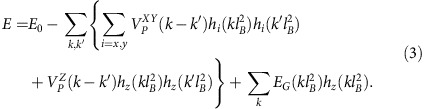


Here 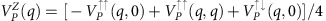 and 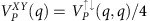 are the interaction coefficients, which characterize the anisotropy of the interactions within a layer and between the layers.

**Figure 3:**
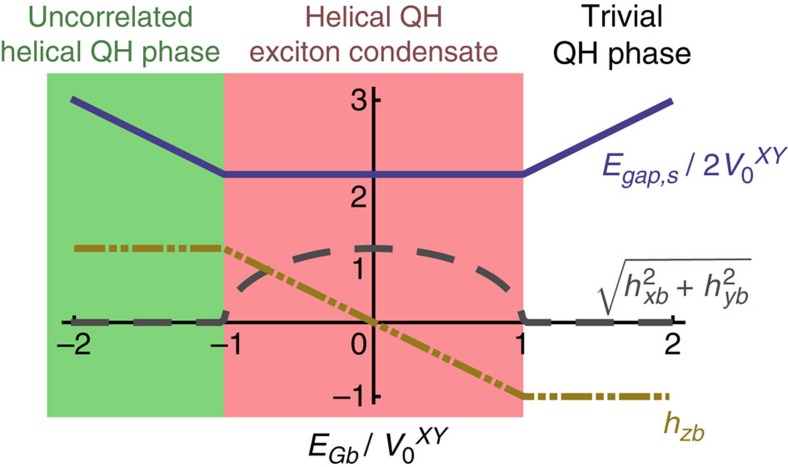
Phase diagram. For 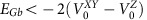 the system supports an uncorrelated helical QH phase. In this phase, there is no spontaneous interlayer phase coherence in the bulk (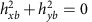) and the system supports helical edge states (guaranteed by spin-resolved Chern numbers 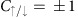). For 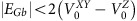 the system supports a helical QH exciton condensate phase. In this phase, there is spontaneous interlayer phase coherence (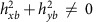), and the system supports exotic confined edge excitations (see below). For 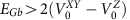 the system is in a trivial QH phase, where the edge is fully gapped. *E*_Gb_ can be controlled with gate voltages or magnetic field (If 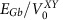 is varied with magnetic field the ratio 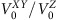 will change within the phase-diagram. Nevertheless, we find that the phase-diagram stays qualitatively similar). Charged bulk excitations are gapped everywhere in the phase diagram (*E*_gap,s_>0). We have chosen 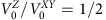.

We start by considering an infinite system. In this case, the pseudospin direction **h**(**r**) is spatially homogeneous. By minimizing the energy functional (3), we find that the pseudospin direction **h**_*b*_ is determined by the parameters *E*_Gb_ and 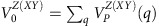 (Supplementary Note 3). Here *E*_Gb_ acts as an effective magnetic field preferring the pseudospin direction along 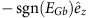. On the other hand, the interactions prefer the pseudospin directions within the (x,y)-plane (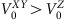), and the energy cost to rotate the pseudospin so that it points along the *z* direction is proportional to 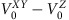. Thus, as a balance between these two competing effects, the direction of the pseudospin is tilted away from the (*x*, *y*)-plane, resulting in the three distinct phases of the system, which are summarized in Fig. 3. For sufficiently large |*E*_Gb_|, we see that |*h*_*zb*_|=1, meaning that only one layer is occupied. The phases *h*_*zb*_=1 (uncorrelated helical QH phase) and *h*_*zb*_=−1 (trivial QH phase) are topologically distinct from each other. For 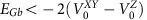 and *h*_*zb*_=1 the system supports helical edge modes (the spin-resolved Chern numbers are 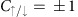). On the other hand, in the regime 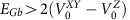 and *h*_*zb*_=−1, the edge is completely gapped (the spin-resolved Chern numbers are 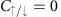). Between these two phases is the helical QH exciton condensate phase, where |*h*_*zb*_|<1 and thus 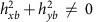. In this phase the direction of the pseudospin projection onto the (*x, y*)-plane is determined spontaneously. Because the pseudospin in this system labels spin and layer index simultaneously, this phase has simultaneously spontaneous in-plane spin polarization and spontaneous interlayer phase coherence.

The bulk gap for single particle excitations *E*_gap,s_ can be calculated using Hartree-Fock linearization (Supplementary Note 3)


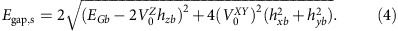


In addition to the single particle excitations, the helical QH exciton condensate supports collective excitations^13^: the neutral pseudospin waves (Goldstone mode) give rise to spin and counterflow charge superfluidity, and the lowest energy charged excitations are topological pseudospin textures, which carry fractional charge 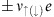. Here 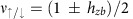 are the pseudospin resolved filling factors of the different Landau levels. The energy required to create these charged excitations is slightly lower than *E*_gap,s_ (ref. 13).

We point out that although *h*_*zb*_=±1 are topologically distinct phases, the bulk gap for creating charged excitations never closes, when one tunes from one phase to the other by controlling *E*_Gb_. This is possible because the pseudospin rotation symmetry, which protects the existence of spin-resolved Chern numbers as topological numbers, is spontaneously broken in the helical exciton condensate phase. The interacting BHZ model for bilayers shows somewhat similar behaviour also at zero magnetic field, where a trivial insulator phase can be connected to a quantum spin Hall insulator phase without closing of the bulk gap, because of an intermediate phase where the time-reversal symmetry is spontaneously broken^27^. It is also experimentally known that the exciton condensate phase with 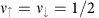 can be smoothly connected to uncorrelated QH state with 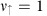 and 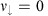 in conventional QH bilayers^28^. Experimental investigations of InAs/GaSb bilayers in the QH regime^18^,^29^ are consistent with this prediction, because no gap closing has been observed as a function of magnetic field.

### Confinement-deconfinement transition of edge excitations

**Figure 4:**
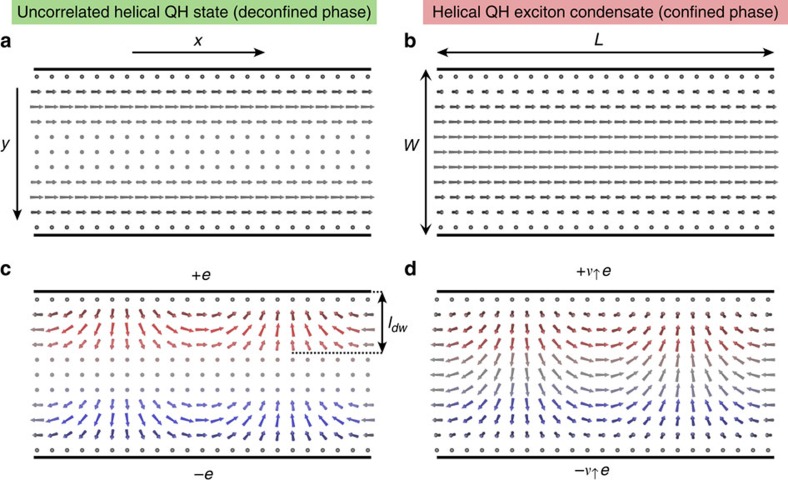
Spin textures. (**a**,**b**) Ground state spin textures for the uncorrelated helical QH phase and the helical QH exciton condensate phase, respectively. In both cases there exists a robust domain wall, where the polar angle of the pseudospin magnetization *θ*(*y*) rotates from π to *θ*_*b*_ along the *y*-direction. In the uncorrelated phase *θ*_b_=0 but in the helical QH exciton condensate phase *θ*_*b*_≠0, *π* indicating spontaneous interlayer phase coherence in the bulk. In both cases the ground states are degenerate for all choices of the constant azimuthal angle ϕ of the pseudospin magnetization. (We have chosen ϕ=0.) (**c**,**d**) Charged excitations can be created by letting the azimuthal angle ϕ(*x*) to rotate along the *x*-direction. In a closed system (obtained by connecting the ends of the sample to form a narrow cylinder) ϕ(*x*) must rotate integer multiples of 2*π*. The energy to create such kind of excitation in the uncorrelated helical QH phase scales as 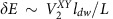, and the elementary excitations, which carry charge ±*e*, can be created independently on the different edges. On the other hand, deep inside the helical QH exciton condensate phase 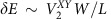 and the elementary charges are 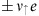. Furthermore, the charged edge excitations are confined: a charge 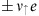 on one of the edges is always connected to the opposite charge on the other edge by a stripe of rotated pseudospins through the bulk, and thus isolated charges cannot be observed at low energies. The charge density obtained from equation (5) is shown with red (positive charge density) and blue (negative charge density) colours.

We now turn to the description of the ground state pseudospin texture *h*_*z*_(*y*)=cos[*θ*_0_(*y*)], *h*_*x*_(*y*)=sin[*θ*_0_(*y*)]cos (*ϕ*) and *h*_*y*_(*y*)=sin[*θ*_0_(*y*)]sin(*ϕ*) at the edge. (The ground state will be degenerate with respect to the choice of *ϕ*.) As discussed above close to the edge *E*_*G*_(*y*)>0 takes large values, because the edge states are topologically protected to exist at all energies between the lowest Landau levels and higher ones. Therefore close to the edge *θ*_0_(*y*)=*π*. On the other hand, in the bulk *θ*_0_(*y*)=*θ*_*b*_=arccos(*h*_*zb*_). This means that there always exists a domain wall, where *θ*_0_ rotates from *π* to *θ*_*b*_. Although the existence of the domain wall is a robust topological property of the system, the detailed shape of *θ*_0_(*y*) and the length scale *l*_*dw*_, where this rotation happens, depend on the details of the sample (Supplementary Note 4). The ground states in the uncorrelated helical QH phase (*θ*_*b*_=0) and helical QH exciton condensate phase (*θ*_*b*_≠0, *π*) are illustrated in Fig. 4a,b, respectively. It turns out that the existence of spontaneous interlayer phase coherence, which distinguishes the two different phases of matter, also has deep consequences on the nature of the low-energy excitations in this system.

By using a Hartree-Fock linearization for the ground state, we find that the single particle excitations are gapped also close to the edge, and the magnitude of the energy gap is determined by the Coulomb energy scale 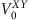. However, similarly as for the case of a coherent domain wall in QH ferromagnetic state in graphene^23^, the lowest energy edge excitations are not the single-particle ones. Namely, the ground state is degenerate with respect to the choice of *ϕ*, and therefore in accordance with the Goldstone's theorem the system supports low-energy excitations described by spatial variation of 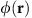. Due to the general relationship between the electric and topological charge densities in QH ferromagnets^13^,^23^ (Supplementary Note 5)


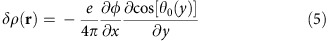


these excitations also carry charge, which is localized at the edges of the sample. In this section we illustrate these excitations in a closed system obtained by connecting the ends of the sample to form a narrow cylinder with width *W* and circumference *L* (Alternatively, instead of comparing the energies to create elementary excitations in a closed system one could compare the energies needed to create a fixed charge density on the edge. This generalization allows the possibility to consider open systems.). We point out that in addition to the topological contribution (5) there can also be non-topological contributions to the electric charge density of pseudospin textures due to excitation of higher Landau levels. However, these contributions are small for the pseudospin textures which are smooth on the scale of *l*_*B*_ (Supplementary Note 8 and Supplementary Fig. 2).

We start by considering this kind of closed system, where 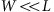 (see Fig. 4). This geometry is topologically equivalent to a Corbino ring, which has been experimentally realized for QH exciton condensates^30^,^31^. Using equations (3) and (5) we find that the lowest energy excitations correspond to rotation of *ϕ*(*x*) by 2*π* and carry a net charge within one of the edges (Supplementary Note 6). They have an energy 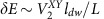 in the uncorrelated phase and 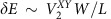 deep in the helical exciton condensate phase. Here 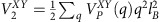 characterizes the cost of exchange energy caused by ∇*ϕ*(*x*) (Supplementary Note 4).

In the uncorrelated phase these excitations have a charge ±*e*. By inspecting Fig. 4, we notice that because the spin points along *z*-direction in the bulk, there is no rotation happening in the bulk. This means that we can choose separate fields *ϕ*_1_(*x*) and *ϕ*_2_(*x*) for the two edges, so that these excitations can be created independently on the different edges much as in graphene^23^.

The situation is dramatically different in the helical QH exciton condensate phase. There, the elementary excitations in a closed system have a charge 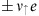. Moreover, as illustrated in Fig. 4, a charge 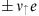 on one of the edges is always connected to the opposite charge on the other edge by a stripe of rotated bulk pseudospins. Breaking the bulk pseudospin configuration costs an energy comparable to the Coulomb energy, and thus isolated charges cannot be observed at low energies. This means that this type of charged edge excitations in the helical QH exciton condensate phase are confined.

**Figure 5:**
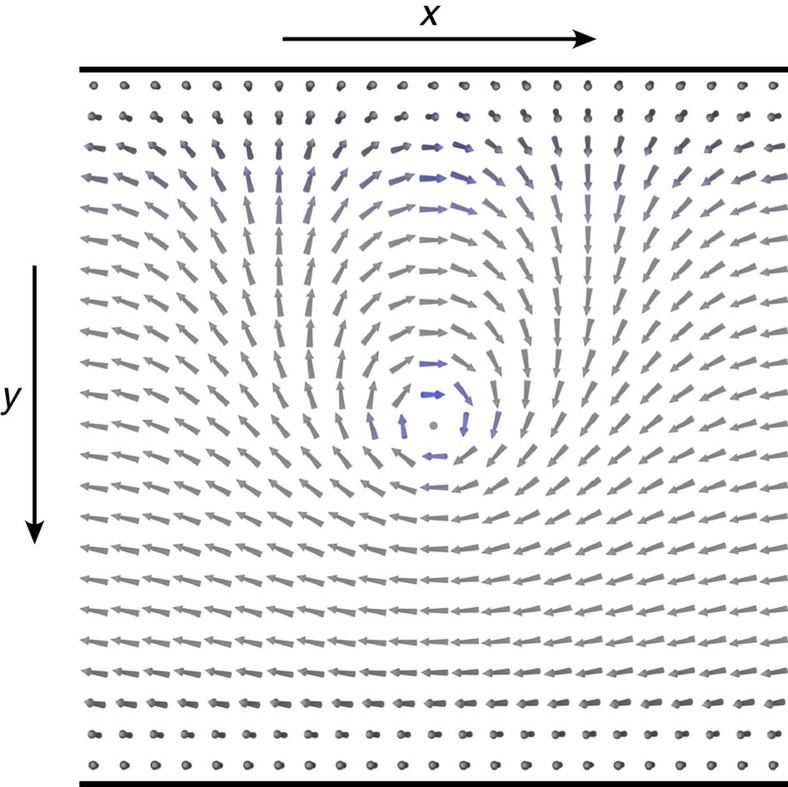
Charged excitation localized at one edge of the sample in the helical QH exciton condensate. The excitation can be visualized as a meron-antimeron pair, where the meron is localized inside the sample and anti-meron—outside. These excitations become the lowest energy charged excitations in sufficiently wide samples (otherwise similar cylinder geometry as in Fig. 4), where it is energetically favourable to break the stripe connecting opposite charges at the different edges by a creation of a bulk meron. Depending on the profile *E*_*G*_(*y*) near the edge, the lowest energy excitation is charge-neutral or having charge ±*e*. The charge is determined by the pseudospin orientation in the center of the meron. The energy of such excitation is of order of Coulomb energy 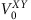.

It is illustrative to consider what happens to the excitations in the helical QH exciton condensate, when the width of the sample is increased. Namely, the excitation energy increases proportionally to the width of the sample 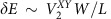 and eventually for *W*∼*L* it becomes energetically favourable instead of having a large area of rotating spins between two edges to create a bulk meron (Fig. 5). This resembles the physics of quarks, where the growing separation of a quark-antiquark pair eventually results in the creation of a new quark-antiquark pair between them.

### Luttinger liquid theory and nonlocal transport

To predict experimentally measurable consequences of the charge confinement, we consider nonlocal transport in an open system. By considering the time-dependent field theory for the pseudospin for a reasonably narrow sample in the helical QH exciton condensate phase we arrive at an effective one-dimensional Hamiltonian (Supplementary Note 7)


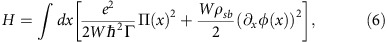


where 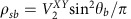 is the pseudospin stiffness and 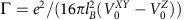 describes the interlayer capacitance per unit area, which is strongly enhanced from the electrostatic value by the exchange interactions. The one-dimensional charge densities in the different edges (labelled 1 and 2) 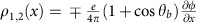 are always opposite and determined by a single field *ϕ*(*x*), highlighting the confinement of the charged edge excitations. The one-dimensional theory describes a Luttinger liquid, and the so-called Luttinger parameter *K* in the convention used in ref. 32, is given by


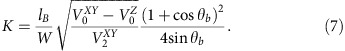


**Figure 6:**
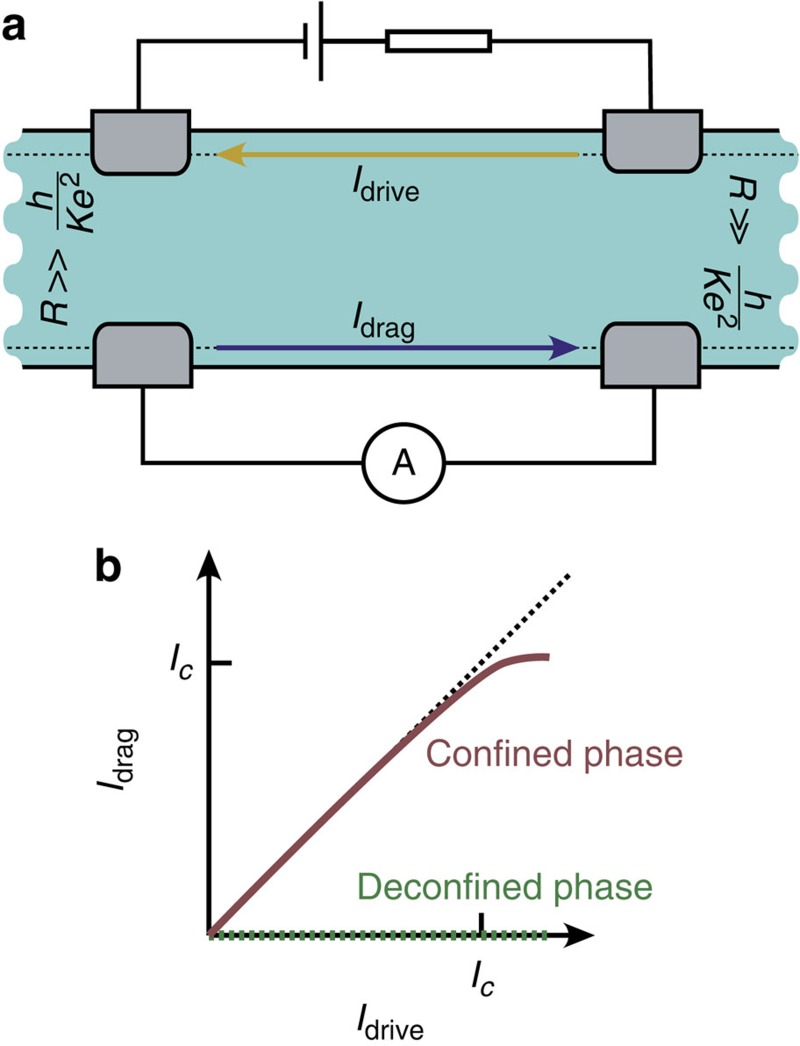
Non-local transport to demonstrate the charge confinement. (**a**) The transport geometry. A drive current is applied on one of the edges and the resulting drag current is measured on the opposite edge. (**b**) Drag current *I*_drag_ as a function of the driving current *I*_drive_ in the geometry from **a**. Due to the charge confinement in the helical QH exciton condensate phase, the drive current necessarily gives rises to an opposite drag current on the other edge. On the other hand, in the uncorrelated (deconfined) phase, there is only a weak drag current due to the Coulomb force acting between the charges.

The Luttinger parameter in quantum Hall systems determines the conductance for ideal contacts *G*_*cf*_=*Ke*^2^/*h* (ref. 32, 33). Because the pseudospin waves are charge neutral in the bulk, conductance decreases with *W* as *G*_*cf*_∝1/*W*. It is important to notice that in this system *G*_*cf*_ describes the conductance for a counterflow/drag geometry, where opposite currents are flowing in the two edges. The helical QH exciton condensate phase does not support net transport current as long as the voltages *eV* are small compared with *ħvπ*/*W* (Supplementary Note 7). This automatically leads to a remarkable transport property that characterizes the helical QH exciton condensate phase. Namely, by considering a non-local transport geometry shown in Fig. 6a, where a drive current is applied on one of the edges and a resulting drag current is measured on the opposite edge, we find that necessarily *I*_drag_=*I*_drive_ at small voltages. This should be contrasted to the uncorrelated helical phase, where the charged edge excitations are deconfined. In that case, one has two independent Luttinger liquid theories for the two edges (Supplementary Note 7), and therefore one expects only a weak drag current due to the Coulomb force acting between the charges. For 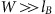, we expect that this effect is negligible compared with drag current in the confined phase.

Finally, to estimate the critical current *I*_*c*_, where the relation *I*_drag_=*I*_drive_ breaks down, we notice that the maximum voltage is determined by the gap *eV*_*max*_≈*ħvπ*/*W*. By using reasonable estimates 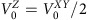, 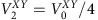, *θ*_*b*_=π/2, *W*=20 *l*_*B*_, *l*_*B*_=10 nm, *v*=14 km s^−1^ (refs 34, 35), we find *I*_*c*_=*G*_*cf*_*V*_*max*_≈0.1 nA.

## Discussion

In summary, we have predicted the existence of a helical QH exciton condensate state in band-inverted electron-hole bilayers. We have shown that the counterpropagating edge modes give rise to a ground state pseudospin texture, where the polar angle of the pseudospin magnetization *θ*(*y*) rotates from the boundary value π to the bulk value *θ*_*b*_ along the direction perpendicular to the edge. Low-energy charged excitations can be created by letting the azimuthal angle of the pseudospin polarization *ϕ*(*x*) to rotate along the edge. Remarkably, in a sufficiently narrow Hall bar these charged edge excitations are confined in the presence of spontaneous interlayer phase-coherence (*θ*_*b*_≠0, *π*): a charge on one of the edges always gives rise to the opposite charge on the other edge, and thus isolated charges cannot be observed at low energies. Moreover, we predict the possibility to control *θ*_*b*_ with a magnetic field and gate voltages. This allows to study a confinement-deconfinement transition, which occurs simultaneously with the bulk phase-transition between the helical QH exciton condensate phase (*θ*_*b*_≠0, *π*) and the uncorrelated helical QH phase (*θ*_*b*_=0).

The helical QH exciton condensate phase can be experimentally probed using Josephson-like interlayer tunnelling and counterflow superfluidity^1^,^8^,^9^,^13^,^30^,^31^,^34^,^35^. Moreover, because the pseudospin in this system describes simultaneously both the spin and the layer degrees of freedom, the helical QH exciton condensate phase can also be probed using the spin superfluidity and the NMR techniques^8^. Perhaps it is even possible to use local probe techniques to image the confinement-deconfinement transition and the confinement physics as illustrated in Figs 4 and 5. Finally, we have shown that the charge confinement also gives rise to a remarkable new transport property. Namely, a drive current applied on one of the edges gives rise to exactly opposite drag current *I*_drag_=*I*_drive_ at the other edge.

Our results for the confinement of the edge excitations may also be applicable to the so-called canted antiferromagnetic phase, which is predicted to appear in graphene^36^. Similarly to the helical QH exciton condensate state considered in this paper, the canted antiferromagnetic phase is characterized by a spontaneously broken U(1)-symmetry in the bulk and a single edge supports only gapped meron-antimeron excitations^36^.

The phenomena of confinement stemming from the particle physics models^24^ has been studied also in condensed matter systems^37^,^38^,^39^,^40^,^41^. However, we expect that the combination of the different techniques for probing the helical QH exciton condensate phase will provide a more intuitive understanding and new perspectives on the confinement physics.

We also point out that InAs/GaSb bilayers is a promising system for superconducting applications, and edge-mode superconductivity has already been experimentally demonstrated in the QSH regime^42^. In the presence of superconducting contacts, the helical QH exciton condensate may provide a new route for realizing exotic non-local Josephson effects and non-Abelian excitations, such as parafermions^5^,^6^.

## Additional information

**How to cite this article:** Pikulin, D. I. *et al*. Confinement-deconfinement transition due to spontaneous symmetry breaking in quantum Hall bilayers. *Nat. Commun.* 7:10462 doi: 10.1038/ncomms10462 (2016).

## Supplementary Material

Supplementary Information Supplementary Figures 1-2, Supplementary Notes 1-8 and Supplementary References

## Figures and Tables

**Figure 1:**
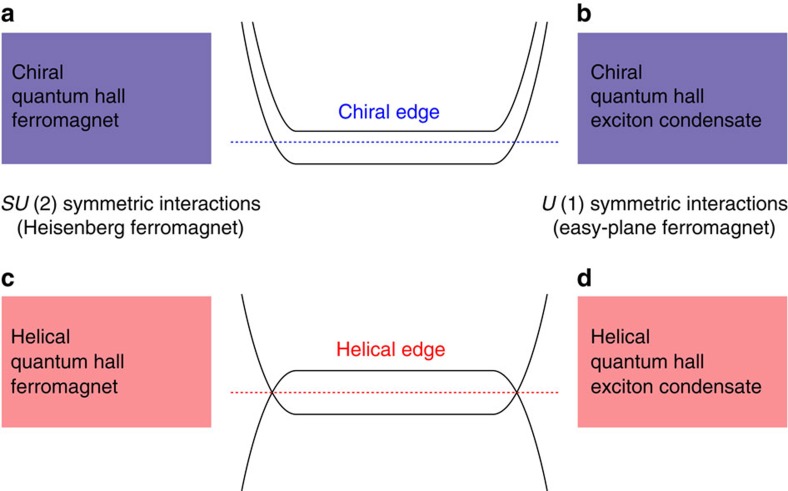
Four different types of QH pseudospin ferromagnetic states at *v*=1. The single layer realizations (**a**,**c**) realize Heisenberg ferromagnets because the interactions have *SU*(2) symmetry. The bilayer QH exciton condensates (**b**,**d**) can be described as an easy-plane ferromagnet with a spontaneously broken *U*(1)-symmetry. We argue that the classification is additionally enriched as the QH systems can support either chiral (**a**,**b**) or helical (**c**,**d**) edge excitations.
