# Supplementary material for: Confinement-deconfinement transition due to spontaneous symmetry breaking in quantum Hall bilayers
Source: Nat Commun. 2016 Jan 25;7:10462. doi: 10.1038/ncomms10462 (PMC4737752; doi:10.1038/ncomms10462)
Supplement: Supplementary Information — Supplementary Figures 1-2, Supplementary Notes 1-8 and Supplementary References [file ncomms10462-s1.pdf]

SUPPLEMENTARY NOTE 1. EFFECTIVE MODEL FROM THE BHZ HAMILTONIAN

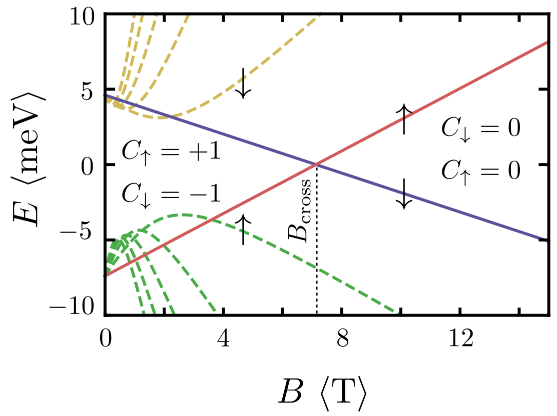

Supplementary Fig. 1. **Landau level fan for the InAs/GaSb bilayer.** The figure shows the energies of the lowest 6 Landau levels on each side of the Fermi level (in terms of the minimum of the absolute value of energy as a function of magnetic field) for the Hamiltonian (S1) with characteristic values of the parameters for InAs/GaSb bilayers $\mathcal{M} = -6$ meV, $\mathcal{C} = -1.4$ meV, $\mathcal{B} = -78.3$ eVÅ^2, $\mathcal{D} = -18$ eVÅ^2 and $\mathcal{A} = 0.62$ eVÅ [6]. We have used the bulk values for the g-factors $g_e = -14.7$ and $g_h = -9.3$. For $B < B_{\rm cross}$ the spin-resolved Chern numbers are $C_\uparrow = -C_\downarrow = 1$ (helical edge modes) whereas for $B > B_{\rm cross}$ the spin-resolved Chern numbers are $C_\uparrow = C_\downarrow = 0$ (trivial insulator).

We consider bilayer QSH systems, such as InAs/GaSb bilayers, described by the BHZ Hamiltonian [1, 2]

$$H = \{\mathcal{M} - \mathcal{B}[(k_x - \frac{y}{l_B^2})^2 + k_y^2]\}\sigma_0\tau_z + \mathcal{A}(k_x - \frac{y}{l_B^2})\sigma_z\tau_x - \mathcal{A}k_y\sigma_0\tau_y + \{\mathcal{C} - \mathcal{D}[(k_x - \frac{y}{l_B^2})^2 + k_y^2]\}\sigma_0\tau_0 + \frac{g_e\mu_B B}{4}\sigma_z(\tau_0 + \tau_z) + \frac{g_h\mu_B B}{4}\sigma_z(\tau_0 - \tau_z), \quad \text{(S1)}$$

where σ and τ are Pauli matrices in spin and electron-hole basis correspondingly, $\mathcal{M}$ describes the distance between the bottoms of electron and hole bands (in the inverted regime $\mathcal{M} < 0$), $\mathcal{C}$ is the chemical potential, $\mathcal{B}, \mathcal{D} < 0$ ($|\mathcal{B}| > |\mathcal{D}|$) determine the effective masses for the electron and hole bands, $g_{e(h)}$ are the g-factors for electron and hole bands, respectively, and the magnetic field has been written in the Landau gauge with $l_B = \sqrt{\hbar/eB}$ being the magnetic length. This Hamiltonian describes an electron-hole bilayer, where the electron band in one of the layers is made out of s-orbitals and the hole band in the other layer is made out of p-orbitals, so that the tunneling between the layers (proportional to $\mathcal{A}$) is odd in momentum. There exist two strategies for constructing this kind of bilayer system. The first possibility is to use two different semiconducting materials where the s-like electron band in one of the materials and p-like hole band in the other material are inverted such as InAs/GaSb bilayers [2–4]. The second strategy is to use in both layers the same semiconductor where the s-like electron band and the p-like hole band are close in energy, so that in a gated device one can reach a situation where the s-like electron band is active close to Fermi energy in one layer whereas the p-like hole band is active in the other. A promising approach to realize this possibility is to construct a bilayer in such a way that each layer individually supports the QSH effect [5]. Here we have neglected the spin-orbit coupling terms arising due to structural and bulk inversion asymmetry. In InAs/GaSb bilayers these terms are estimated to be very small [6]. The Landau level spectrum for InAs/GaSb bilayers is shown in Supplementary Fig. 1. In this material the parameters of the model can be tuned with the help of gate voltages and widths of the quantum wells. Here we have used characteristic values of the parameters [6] and the bulk values for the g-factors.

The two lowest Landau level wavefunctions for this model are

$$\psi_{k;\uparrow(\downarrow)} = \frac{e^{ikx}}{\sqrt{Ll_B}}\phi\left(\frac{y - kl_B^2}{l_B}\right)\left(1(0) \;\; 0 \;\; 0 \;\; 0(1)\right)^T, \quad \text{(S2)}$$

where $\phi(\xi) = e^{-\xi^2/2}/\pi^{1/4}$. Notice that spin and orbital degrees of freedom are locked, so that the pseudospin $\uparrow$ ($\downarrow$) means simultaneously up (down) spin and upper (lower) layer. This locking is caused by the tunneling term proportional to $\mathcal{A}$, which is linear in momentum. Due to the existence of this term the electron-like Landau level with spin down (hole-like Landau level with spin up) couples to a hole-like (electron-like) higher Landau level, and as

a result of this coupling these Landau levels are well separated in energy from the Landau levels given by Eq. (S2). Within the subspace generated by these wave functions, the single-particle Hamiltonian is

$$\hat{H}_0 = \sum_k [\hat{\psi}^\dagger_{k,\uparrow}\hat{\psi}_{k,\uparrow} - \hat{\psi}^\dagger_{k,\downarrow}\hat{\psi}_{k,\downarrow}] E_G(kl_B^2). \tag{S3}$$

Here $\hat{\psi}^\dagger_{k,\uparrow(\downarrow)}$ and $\hat{\psi}_{k,\uparrow(\downarrow)}$ are the creation and annihilation operators corresponding to the electronic states described by Eq. (S2), and we have fixed the chemical potential so that the total density corresponds to one of these Landau levels being filled and the other empty ($\nu_T = \nu_\uparrow + \nu_\downarrow = 1$). The Fermi level is set to be at zero energy. For an infinite system in y-direction, the energy E_G is independent on momentum and given by $E_{Gb} = \mathcal{M} - \frac{e\mathcal{B}}{\hbar}B + \frac{g_e+g_h}{4}\mu_B B$. Due to the presence of an edge the Landau levels obtain an energy-momentum dispersion. According to Eq. (S2) the momentum is directly connected to the position y in real space, so that this energy-momentum dispersion can also be written as a position-dependent energy $E_G(y) = E_G(kl_B^2)$. The Landau level originating from the electron (hole) band always disperses upwards (downwards) in energy, when approaching the edge. Moreover, because the edge states exist at all energies between the lowest Landau levels and the higher ones, close to the edge $E_G(y) > 0$ reaches extremely large values, which are on the same order as the energy separation between the bulk Landau levels. The spatial variation of $E_G(y)$ occurs within a characteristic length scale l_0, which for clean sharp edge is given by $l_0 \sim l_B$. However, because the edge state velocity $v_s = \frac{1}{\hbar}\frac{dE_G}{dk}$ in the non-interacting theory is directly related to l_0, the edge roughness and disorder renormalize l_0 upwards (v_s downwards), and hence l_0 can be considerably larger than l_B. The explicit form of the wave functions is given by Eq. (S2) only if $l_0 \gg l_B$, but our main results are expected to remain valid even if this condition is not satisfied.

Assuming that there is a band inversion at zero magnetic field ($\mathcal{M}, \mathcal{B} < 0$), there is a crossing of the lowest Landau levels at magnetic field $B_{\text{cross}} = \mathcal{M}/(\frac{e\mathcal{B}}{\hbar} - \frac{g_e+g_h}{4}\mu_B)$, where the band inversion is removed. For $B < B_{\text{cross}}$, we notice that $E_G(y) = E_{Gb} < 0$ in the bulk but $E_G(y) > 0$ close to the edge, yielding helical edge states. On the other hand for $B > B_{\text{cross}}$, $E_G(y) > 0$ everywhere, and therefore the edge is gapped according to the non-interacting theory. The change in the edge structures shows up in the spin resolved Chern numbers. Namely for $B < B_{\text{cross}}$ the spin-resolved Chern numbers are $C_\uparrow = -C_\downarrow = 1$ (helical edge modes), whereas for $B > B_{\text{cross}}$ $C_\uparrow = C_\downarrow = 0$ (trivial insulator).

SUPPLEMENTARY NOTE 2. ENERGY FUNCTIONAL

Within the subspace generated by the lowest Landau level wave functions, the projected Hamiltonian can be written as $\hat{H} = \hat{H}_0 + \hat{H}_I$, where the interactions are described by

$$\hat{H}_I = \frac{1}{2}\sum_{\sigma,\sigma'}\sum_{k,k',q} V_P^{\sigma\sigma'}(k-k',q)\hat{\psi}^\dagger_{k\sigma}\hat{\psi}^\dagger_{k',\sigma'}\hat{\psi}_{k'+q\sigma'}\hat{\psi}_{k-q\sigma}. \tag{S4}$$

Here, the projected Coulomb interactions can be written as

$$V_P^{\sigma\sigma'}(k-k',q) = \frac{\pi^{1/4}}{\sqrt{2}}\phi_0^2\left(\frac{ql_B}{\sqrt{2}}\right)\frac{1}{Ll_B}\int d^2r\ V^{\sigma\sigma'}(\mathbf{r})e^{iqx}\phi_0\left(\frac{y-(k-k'-q)l_B^2}{l_B}\right). \tag{S5}$$

To simplify the expressions we assume that the quantum wells are very narrow so that

$$V^{\uparrow\uparrow}(\mathbf{r}) = V^{\downarrow\downarrow}(\mathbf{r}) = \frac{e^2}{4\pi\epsilon\epsilon_0 r}, \qquad V^{\uparrow\downarrow}(\mathbf{r}) = V^{\downarrow\uparrow}(\mathbf{r}) = \frac{e^2}{4\pi\epsilon\epsilon_0\sqrt{r^2+d^2}}. \tag{S6}$$

This assumption does not change the results qualitatively. However, one should keep in mind that quantitatively the energy scales associated with the interaction effects are overestimated, because the finite width of the quantum well would reduce the effective interaction strengths.

To compute the energy for a pseudospin texture we follow closely the approach developed in Ref. 7. We assume that the components of the pseudospin $h_i(y) = h_i(kl_B^2)$ are slowly varying with respect to l_B, so that we can express the many particle wave function as

$$|\Psi[\mathbf{h}(kl_B^2)]\rangle = \prod_k \frac{1}{\sqrt{2[1-h_z(kl_B^2)]}}\left\{\left[h_x(kl_B^2) - ih_y(kl_B^2)\right]\hat{\psi}^\dagger_{k,\uparrow} + \left[1-h_z(kl_B^2)\right]\hat{\psi}^\dagger_{k,\downarrow}\right\}|0\rangle. \tag{S7}$$

Here $\sum_i h_i^2(kl_B^2) = 1$. The energy functional can be obtained by calculating

$$E\big[\mathbf{h}(kl_B^2)\big] = \langle\Psi\big[\mathbf{h}(kl_B^2)\big]|\hat{H}_0 + \hat{H}_I|\Psi\big[\mathbf{h}(kl_B^2)\big]\rangle. \tag{S8}$$

Using the Wick's theorem, we obtain

$$E\big[\mathbf{h}(kl_B^2)\big] = E_0 + \sum_k E_G(kl_B^2) h_z(kl_B^2) - \sum_{k,k'} \left\{ V_P^Z(k-k') h_z(kl_B^2) h_z(k'l_B^2) + \sum_{i=x,y} V_P^{XY}(k-k') h_i(kl_B^2) h_i(k'l_B^2) \right\}, \quad \text{(S9)}$$

where

$$V_P^Z(q) = \frac{1}{4}\Big[-V_P^{\uparrow\uparrow}(q,0) + V_P^{\uparrow\uparrow}(q,q) + V_P^{\uparrow\downarrow}(q,0) \Big] = -\frac{V_C}{4\sqrt{2\pi}L} \int_{-\infty}^{\infty} dy \ln\left[\frac{y^2+d^2}{y^2}\right] e^{-(y/l_B - ql_B)^2/2} + \frac{V_C}{4\sqrt{2\pi}L} e^{-q^2 l_B^2/2} \int_{-\infty}^{\infty} dx\, e^{iqx} e^{x^2/4l_B^2} K_0\left(\frac{x^2}{4l_B^2}\right) \quad \text{(S10)}$$

and

$$V_P^{XY}(q) = \frac{1}{4} V_P^{\uparrow\downarrow}(q,q) = \frac{e^{-q^2 l_B^2/2} V_C}{4\sqrt{2\pi}L} \int_{-\infty}^{\infty} dx\, e^{iqx} e^{x^2/4l_B^2 + d^2/4l_B^2} K_0\left(\frac{x^2+d^2}{4l_B^2}\right). \quad \text{(S11)}$$

Here the characteristic energy scale of the Coulomb interactions is $V_C = e^2/(4\pi\epsilon\epsilon_0 l_B)$.

SUPPLEMENTARY NOTE 3. MEAN FIELD SOLUTIONS IN THE BULK

Before describing the pseudospin texture at the edge, let's solve the ground state orientation of the pseudospin in the bulk. By assuming a homogeneous solution in the bulk, we obtain

$$E[\mathbf{h}] = E_0 + \frac{L^2}{2\pi l_B^2}\Big[E_{Gb} h_z - V_0^Z h_z^2 - V_0^{XY}(h_x^2 + h_y^2) \Big], \quad \text{(S12)}$$

where E_{Gb} is the bulk value of $E_G(y)$ (in the inverted regime $E_{Gb} < 0$ and in the non-inverted regime $E_{Gb} > 0$),

$$V_0^Z = \sum_q V_P^Z(q) = V_C \frac{1}{4}\left[\sqrt{\frac{\pi}{2}} - \frac{d}{l_B}\right] \quad \text{(S13)}$$

and

$$V_0^{XY} = \sum_q V_P^{XY}(q) = V_C \frac{1}{4}\sqrt{\frac{\pi}{2}} e^{d^2/2l_B^2} \mathrm{Erfc}\left[\frac{d}{\sqrt{2}l_B}\right]. \quad \text{(S14)}$$

By minimizing the energy given Eq. (S12) with a constraint $\sum_i h_i^2 = 1$, we obtain

$$h_{zb} = \begin{cases} 1, & -\frac{E_{Gb}}{2(V_0^{XY} - V_0^Z)} > 1 \\ -\frac{E_{Gb}}{2(V_0^{XY} - V_0^Z)}, & \left|\frac{E_{Gb}}{2(V_0^{XY} - V_0^Z)}\right| < 1 \\ -1, & -\frac{E_{Gb}}{2(V_0^{XY} - V_0^Z)} < -1 \end{cases} \quad \text{(S15)}$$

The other components satisfy $h_{xb}^2 + h_{yb}^2 = 1 - h_{zb}^2$ and the energy is degenerate with respect to the rotations in the (x,y)-plane.

To estimate the single particle excitation gap in the bulk, we can construct a mean field Hamiltonian by Hartree-Fock linearization of the interaction terms. This way we obtain

$$\hat{H}_{\text{mf}} = \sum_k (\hat{\psi}_{k,\uparrow}^\dagger, \hat{\psi}_{k,\downarrow}^\dagger) \begin{pmatrix} E_{Gb} + m_{zb} & m_{xb} - im_{yb} \\ m_{xb} + im_{yb} & -E_{Gb} - m_{zb} \end{pmatrix} \begin{pmatrix} \hat{\psi}_{k,\uparrow} \\ \hat{\psi}_{k,\downarrow} \end{pmatrix}, \quad \text{(S16)}$$

where $m_{zb} = -2V_0^Z h_{zb}$, $m_{xb} = -2V_0^{XY} h_{xb}$ and $m_{yb} = -2V_0^{XY} h_{yb}$. The bulk gap for single particle excitations is thus

$$E_{\text{gap,s}} = 2\sqrt{(E_{Gb} - 2V_0^Z h_{zb})^2 + 4(V_0^{XY})^2 (h_{xb}^2 + h_{yb}^2)}. \quad \text{(S17)}$$

In addition to the crossing of the Landau levels as a function of magnetic field, several other conditions need to be satisfied in order to realize the helical exciton condensate phase: (i) The other Landau levels at the crossing point are

separated in energy so that they are not excited. (ii) The densities close to the charge neutrality point can be obtained so that $\nu_\uparrow$ and $\nu_\downarrow$ can be controlled with magnetic field and gate voltages. (iii) The layer separation described by d/l_B can be made sufficiently small to reach the exciton condensate phase. (iv) The temperature can be made small enough to reach the exciton condensate phase. (v) The disorder should not be too strong.

(i) As demonstrated in Supplementary Fig. 1 the typical energy gap to higher Landau levels in InAs/GaSb bilayers is on the order of 10 meV, which is significantly larger than the gap opened by the exciton condensate order parameter. We also point out that although the large gap to higher Landau levels simplifies the theoretical analysis, it may not be necessary for the existence of exciton condensate state because the interaction effects actually tend to enhance this energy gap further. In fact, in GaAs bilayers the lowest Landau levels are separated from the higher ones by the Zeeman energy which is a rather small energy scale, but nevertheless the spin is fully polarized in the exciton condensate state [8–11].

(ii) In InAs/GaSb bilayers the densities close to the charge neutrality point can be experimentally reached in gated devices both in the absence [3, 4, 12, 13] and in the presence [14] of the magnetic field.

(iii) In GaAs quantum Hall bilayers it is known that the exciton condensate phase appears for $d/l_B \lesssim 1.8$ [15–19]. Using the typical parameters of the experimental samples [3, 4, 12] we find that this condition can be easily satisfied in InAs/GaSb bilayers.

(iv) The temperature needs to be smaller than the energy gap opened by the exciton condensate order parameter. Moreover, since we are studying a two dimensional system the actual transition to the exciton condensate phase is a Berezinskii-Kosterlitz-Thouless transition [7], and the transition temperature can be estimated to be on the order of Kelvin. The transition temperatures measured in GaAs bilayers are consistent with this type of estimate [18].

(v) The mean free path should be long compared to the coherence length. In quantum Hall exciton condensates the coherence length is on the order of l_B and therefore this condition is easily satisfied. However, the disorder plays also another role in this system. Namely, the vortices are charged and therefore they can be nucleated by a sufficiently strong disorder potential [20, 21]. Although the detailed consideration of the disorder is not the subject of this paper, we point out that most of the phenomenology of the quantum Hall exciton condensate state survives at least approximately also in the presence of the disorder-nucleated vortices [22–26].

SUPPLEMENTARY NOTE 4. DOMAIN WALL AT THE EDGE

In order to describe the domain wall at the edge we first write $h_z(kl_B^2) = \cos[\theta(kl_B^2)]$, $h_x(kl_B^2) = \sin[\theta(kl_B^2)]$ and $h_y(kl_B^2) = 0$. [There is a degeneracy in (h_x, h_y)-plane, so that we can choose a specific direction arbitrarily.] The energy functional [Eq. (S9)] can then be written as

$$E\big[\theta(kl_B^2)\big] = \sum_k E_G(kl_B^2)\cos[\theta(kl_B^2)] - \sum_{k,k'}\Big\{V_P^Z(k-k')\cos[\theta(kl_B^2)]\cos[\theta(k'l_B^2)] + V_P^{XY}(k-k')\sin[\theta(kl_B^2)]\sin[\theta(k'l_B^2)]\Big\}. \tag{S18}$$

By minimizing this with respect to $\theta(kl_B^2)$, we obtain

$$\tan(\theta(kl_B^2)) = \frac{2\sum_{k'} V_P^{XY}(k-k')\sin[\theta(k'l_B^2)]}{2\sum_{k'} V_P^Z(k-k')\cos[\theta(k'l_B^2)] - E_G(kl_B^2)}. \tag{S19}$$

This can be solved numerically by iterations.

Close to the edge $E_G(y) > 0$ takes large values, because the edge states are topologically protected to exist at all energies between the lowest Landau levels and higher ones. Therefore close to the edge $\theta(y) = \pi$. On the other hand, in the bulk $\theta(y) = \theta_b = \arccos(h_{zb})$. This means the existence of a domain wall, where θ rotates from π to θ_b, is a robust topological property of the system, which is not sensitive to the details of the sample.

The width of the domain wall is $l_{dw} \sim \max\{l_s, l_0\}$, where l_0 is the length scale where $E_G(y)$ changes in the vicinity of the edge and l_s is an intrinsic length scale, which is determined by the balance between the energy gain obtained by rotating θ and the corresponding loss of the exchange energy. A rough estimate is obtained by expanding Eq. (S12) around θ_b and noticing that the loss of exchange energy is approximately determined by (see Section SUPPLEMENTARY NOTE 5)

$$V_2^{XY} = \frac{1}{2}\sum_q V_P^{XY}(q) q^2 l_B^2 = V_C \frac{1}{16}\sqrt{\frac{\pi}{2}} e^{d^2/2l_B^2}\,\mathrm{Erfc}\left[\frac{d}{\sqrt{2}l_B}\right]. \tag{S20}$$

This leads to a simple estimate

$$l_s \approx \sqrt{\frac{2V_2^{XY}}{2(V_0^{XY} - V_0^Z)(1-2h_{zb}^2) - E_{Gb}h_{zb}}}\, l_B. \tag{S21}$$

This length scale l_s diverges at the phase transitions $h_z = 1$, $E_{Gb} = -2(V_0^{XY} - V_0^Z)$ and $h_z = -1$, $E_{Gb} = 2(V_0^{XY} - V_0^Z)$ indicating that $\theta(y)$ approaches the bulk value in a power-law like fashion (outside the phase transitions it approaches the bulk value exponentially). Deep inside the helical quantum Hall exciton condensate phase $l_s \sim l_B$.

SUPPLEMENTARY NOTE 5. CHARGED EDGE EXCITATIONS

We now consider excitations $h_z(\mathbf{r}) = h_z^0(y) + \delta h_z(\mathbf{r})$, $h_x(\mathbf{r}) = \sqrt{1 - h_z^2(\mathbf{r})}\cos[\phi(\mathbf{r})]$ and $h_y(\mathbf{r}) = \sqrt{1 - h_z^2(\mathbf{r})}\sin[\phi(\mathbf{r})]$, which are described by slow spatial variation of the pseudospin within the (x, y)-plane $[\phi(\mathbf{r})]$ and small fluctuations $[\delta h_z(\mathbf{r})]$ of the z-component around the ground state configuration $h_z^0(y) = \cos[\theta_0(y)]$.

For this purpose, we first write Eq. (S9)

$$E\big[\mathbf{h}(kl_B^2)\big] = E_0[h_z(kl_B^2)] - \sum_{k,k'}\bigg\{\sum_{i=x,y} V_P^{XY}(k-k')h_i(kl_B^2)h_i(k'l_B^2)\bigg\}. \tag{S22}$$

By expanding $(i = x, y)$

$$h_i(kl_B^2)h_i(k'l_B^2) = h_i^2(kl_B^2) + h_i(kl_B^2)(k'l_B^2 - kl_B^2)h_i'(kl_B^2) + \frac{1}{2}h_i(kl_B^2)(k'l_B^2 - kl_B^2)^2 h_i''(kl_B^2)... \tag{S23}$$

we obtain

$$\begin{aligned} E\big[\mathbf{h}\big] &= E_0[h_z] - \frac{L}{2\pi l_B^2}\int dy\bigg\{V_0^{XY}\sum_{i=x,y} h_i^2(y) + V_2^{XY} l_B^2 \sum_{i=x,y} h_i(y)h_i''(y)\bigg\} \\ &= E_0[h_z] - \frac{1}{2\pi l_B^2}\int d^2r\bigg\{V_0^{XY}\sum_{i=x,y} h_i^2(\mathbf{r}) + V_2^{XY} l_B^2 \sum_{i=x,y} h_i(\mathbf{r})\nabla^2 h_x(\mathbf{r})\bigg\} \\ &= E_0[h_z] + \frac{1}{2\pi l_B^2}\int d^2r\bigg\{V_2^{XY} l_B^2 \sum_{i=x,y}\big[\nabla h_i(\mathbf{r})\big]^2 - V_0^{XY}\sum_{i=x,y} h_i^2(\mathbf{r})\bigg\}. \end{aligned} \tag{S24}$$

Here, we have transformed from momentum space k to real space y by using $y = kl_B^2$, restored the possibility that $\mathbf{h}(\mathbf{r})$ may also vary in the x-direction (using rotational symmetry of the system), assumed a boundary condition

$$\int d^2r\ \nabla\cdot(h_i\nabla h_i) = 0 \tag{S25}$$

and V_2^{XY} is given by Eq. (S20).

Similar gradient expansion cannot be done for $h_z(\mathbf{r})$ because of the long-range Hartree interactions. However, deep inside the phases $\delta h_z(\mathbf{r})$ mode is gapped by a large energy gap, and therefore we assume that as a first approximation it is enough to take the Hartree interactions into account only via the V_0^Z term. In order to calculate the excitation energy we define a functional

$$E\big[\mathbf{h}, \lambda\big] = E\big[\mathbf{h}\big] + \frac{1}{2\pi l_B^2}\int d^2r\ \lambda(\mathbf{r})(1 - \sum_i h_i(\mathbf{r})^2), \tag{S26}$$

where we have now explicitly implemented the constraint $\sum_i h_i^2 = 1$ with the help of Lagrange multiplier. The minimization of $E\big[\mathbf{h}, \lambda\big]$ with respect to all variables leads to a saddle point equation (S19) and allows us to consider fluctuations $\delta h_i(\mathbf{r})$ around the saddle point independently on each other. For general $E_G(y)$ the saddle point equation can be solved only numerically. Here we proceed by assuming that $E_G(y)$ is slowly varying. This is always satisfied in the bulk and we will comment the influence of the edge corrections below.

With these approximation the excitation energy (energy difference compared to the ground state) can be written as

$$\delta E\big[\delta h_z(\mathbf{r}), \phi(\mathbf{r})\big] = \frac{1}{2\pi l_B^2}\int d^2r\bigg\{V_2^{XY} l_B^2 \sin^2[\theta_0(y)]\big[\nabla\phi(\mathbf{r})\big]^2 + \bigg[(V_0^{XY} - V_0^Z)\sin^2[\theta_0(y)] - \frac{E_G(y)}{2}\cos[\theta_0(y)]\bigg]\delta h_z^2(\mathbf{r})\bigg\}. \tag{S27}$$

A variational wave function describing a general pseudospin texture $\mathbf{h}(\mathbf{r})$ is no longer restricted to the lowest Landau level. Therefore, the many particle wave function needs to be projected to the lowest Landau level with an operator

$\mathcal{P}$, which can be implemented as explained in Ref. 7. This results in a charge density for such kind of excitations, which can be computed from the general relationship between the electric and topological charge densities [7]

$$\delta\rho(\mathbf{r}) = -\frac{e}{8\pi}\epsilon_{\mu\nu}\mathbf{h}(\mathbf{r})\cdot[\partial_\mu\mathbf{h}(\mathbf{r})\times\partial_\nu\mathbf{h}(\mathbf{r})]. \tag{S28}$$

For the low-energy excitations, the charge density is therefore (see also Section SUPPLEMENTARY NOTE 8)

$$\delta\rho(\mathbf{r}) = -\frac{e}{4\pi}\frac{\partial\phi}{\partial x}\frac{\partial\cos[\theta_0(y)]}{\partial y}. \tag{S29}$$

From this expression we see that the charge density is localized in the vicinity of the edge, and the length scale in y-direction is determined by the domain wall width l_{dw}. Moreover, the low-energy charged excitations are always associated with spatial variation of ϕ along the edge, so that the charge density is proportional to $\partial_x\phi$. We will study the nature of these excitations separately in the two opposite limits $E_{Gb} \ll -2(V_0^{XY}-V_0^Z)$ (deep inside the uncorrelated helical quantum Hall state) and $|E_{Gb}| \ll 2(V_0^{XY}-V_0^Z)$ (deep inside the helical quantum Hall exciton condensate phase).

SUPPLEMENTARY NOTE 6. CHARGED EDGE EXCITATIONS IN A CLOSED SYSTEM

We consider narrow quantum Hall systems with width W much smaller than the length L (similarly as shown in Fig. 4 in the main text), and we assume that the system is closed by connecting the ends of the sample. Based on Eqs. (S27) and (S29) we argue that the lowest energy charged excitations are obtained by letting azimuthal angle $\phi(x)$ rotate along the x-direction. In a closed system the angle $\phi(x)$ needs to rotate integer multiple of 2π. The lowest energy excitations in a closed system, which carry a net charge within one of the edges correspond to rotation of $\phi(x)$ by 2π, and they have an energy $\delta E \sim V_2^{XY} l_{dw}/L$ deep inside the uncorrelated phase and $\delta E \sim V_2^{XY} W/L$ deep inside the helical exciton condensate phase.

In the uncorrelated phase we obtain from Eq. (S29) that these excitations have a charge $\pm e$. Moreover, they are deconfined: One can create these excitations independently on the different edges.

In the helical quantum Hall exciton condensate phase, we obtain from Eq. (S29), that the elementary excitations in a closed system have a charge is $\pm\nu_\uparrow e$, where $\nu_\uparrow = (1+\cos\theta_b)/2$. Moreover, a charge $\pm\nu_\uparrow e$ on one of the edges is always connected to the opposite charge on the other edge by a stripe of rotated pseuspins through the bulk. Creating this stripe costs an energy $\delta E \sim V_2^{XY} W/L$, which is proportional to the distance W between the charges. However, breaking the stripe somewhere in the bulk would cost a much larger energy comparable to the Coulomb energy. Thus isolated charges cannot be observed at low energies. This means that in the helical quantum Hall exciton condensate phase the charged edge excitations are confined.

SUPPLEMENTARY NOTE 7. LUTTINGER LIQUID THEORY

To develop time-dependent theory for these excitations, we use the known result that the Euclidian action in the adiabatic approximation can be written as [7]

$$S^E[\mathbf{h},\lambda] = \int_0^\beta d\tau\Big\{\int d^2r\Big[-i\frac{1}{4\pi l_B^2}\mathbf{A}(\mathbf{h})\cdot\partial_\tau\mathbf{h}\Big] + E\big[\mathbf{h},\lambda\big]\Big\}, \tag{S30}$$

where $\nabla_{\mathbf{h}}\times\mathbf{A}(\mathbf{h}) = \mathbf{h}$. Thus by assuming that $E_G(y)$ is slowly varying function, we can expand the action around the saddle point and obtain

$$\delta S^E[\phi,\delta h_z] = \frac{1}{2\pi l_B^2}\int_0^\beta d\tau\int d^2r\Big\{\frac{i}{2}\delta h_z\frac{\partial\phi}{\partial\tau} + V_2^{XY} l_B^2\sin^2\theta_0 + \Big[(V_0^{XY}-V_0^Z)\sin^2\theta_0(y) - \frac{E_G(y)}{2}\cos\theta_0(y)\Big]\delta h_z^2\Big\}. \tag{S31}$$

A. Confined phase

Deep inside the confined phase, all the properties of the system will be determined by the bulk (see below). Therefore, we neglect the effects arising in the vicinity of the edge, and use Eq. (S50) to rewrite the action as

$$\delta S^E[\phi,\delta h_z] = \frac{1}{2\pi l_B^2}\int_0^\beta d\tau\int d^2r\Big\{\frac{i}{2}\delta h_z\frac{\partial\phi}{\partial\tau} + V_2^{XY} l_B^2\sin^2\theta_0 + (V_0^{XY}-V_0^Z)\delta h_z^2\Big\} \tag{S32}$$

We integrate out the massive fluctuations δh_z. This way we obtain

$$S^E[\phi(\mathbf{r},\tau)] = \frac{1}{2\pi l_B^2}\int_0^\beta d\tau \int d^2r \Big\{ \frac{(\partial_\tau \phi)^2}{16(V_0^{XY}-V_0^Z)} + V_2^{XY} l_B^2 \sin^2[\theta_0(y)] \left[\nabla\phi(\mathbf{r})\right]^2 \Big\}. \quad \text{(S33)}$$

By going to real time, we identify the Langrangian density as

$$\mathcal{L} = \frac{\hbar^2\Gamma}{2e^2}\dot{\phi}^2 - \frac{\rho_s(y)}{2}(\nabla\phi)^2. \quad \text{(S34)}$$

Here $\rho_s(y) = V_2^{XY}\sin^2[\theta_0(y)]/\pi$ is the pseudospin stiffness and $\Gamma = e^2/(16\pi l_B^2(V_0^{XY}-V_0^Z))$ describes the interlayer capacitance per unit area, which is strongly enhanced from the electrostatic value by the exchange interactions. (As an important check we notice that if one neglects the exchange contributions in $V_0^{XY}-V_0^Z$, the expression for Γ simplifies to the familiar electrostatic formula for the interlayer capacitance.)

The corresponding equation of motion is

$$\frac{\hbar^2\Gamma}{e^2}\ddot{\phi} - \nabla\left[\rho_s(y)\nabla\phi\right] = 0. \quad \text{(S35)}$$

This equation is translationary invariant in x and t thus we can express the solution as

$$\phi = \phi_0 + \sum_{n,k} e^{-i[\omega_n(k)t-kx]} f_{n,k}(y), \quad \text{(S36)}$$

where ϕ_0 is an arbitrary constant and $f_{n,k}(y)$ describe the different transverse modes with energy-momentum dispersions $E_n(k) = \hbar\omega_n(k)$. By substituting the trial solution (S36) to Eq. (S35), we find that the eigenfunctions $f_{n,k}(y)$ and eigenfrequencies $\omega_n(k)$ satisfy an eigenvalue equation

$$-\frac{\hbar^2\Gamma\omega_n(k)^2}{e^2} f_{n,k}(y) + k^2\rho_s(y) f_{n,k}(y) - \rho_s(y) f''_{n,k}(y) - \rho'_s(y) f'_{n,k}(y) = 0, \quad \text{(S37)}$$

which can be solved numerically.

Numerical results for the helical quantum Hall exciton condensate phase show that the lowest energy mode $f_{0,k}(y)$ can be approximated as a constant throughout the sample. Moreover, the lowest energy mode at reasonably small momentum is separated from higher modes by an energy $\Delta E \sim \hbar v\pi/W$. We restrict the analysis to sufficiently low-energies that these higher modes are not excited. Then we assume that ϕ is constant in y-direction and the dispersion relation is

$$\omega(k) = \sqrt{\frac{e^2\rho_{sb}}{\hbar^2\Gamma}}\,k = vk, \quad \text{(S38)}$$

where ρ_{sb} is the bulk value of $\rho_s(y)$. The Hamiltonian of the system then becomes

$$H = \int dx \left[\frac{e^2}{2W\hbar^2\Gamma}\Pi(x)^2 + \frac{W\rho_{sb}}{2}(\partial_x\phi(x))^2\right], \quad \text{(S39)}$$

where $\Pi(x)$ is the momentum conjugate to $\phi(x)$. The one-dimensional charge densities in the different edges (labelled 1 and 2) are

$$\rho_{1,2}(x) = \mp\frac{e}{4\pi}(1+\cos\theta_b)\frac{\partial\phi}{\partial x}, \quad \text{(S40)}$$

where θ_b is the bulk value of $\theta(y)$. The confinement of the edge excitation clearly shows up here as the the charge densities on the different edges are always opposite and determined by a single field $\phi(x)$. This Hamiltonian describes a Luttinger liquid. In the standard convention [27] the Luttinger liquid theory is written as

$$H = \frac{\hbar}{2\pi}\int dx\left[\frac{vK\pi^2}{\hbar^2}\tilde{\Pi}(x)^2 + \frac{v}{K}(\partial_x\tilde{\phi})^2\right], \qquad \rho(x) = -\frac{e}{\pi}\frac{\partial\tilde{\phi}}{\partial x}. \quad \text{(S41)}$$

To rewrite our Hamiltonian in this convention, we apply the transformation preserving canonical commutation relations

$$\phi(x) = \frac{4}{1+\cos\theta_b}\tilde{\phi}(x), \qquad \Pi(x) = \frac{1+\cos\theta_b}{4}\tilde{\Pi}(x), \quad \text{(S42)}$$

and notice that the charge density at each edge is $\rho_{1,2}(x) = \pm\rho(x)$.

This way, we can identify the Luttinger liquid parameters K and v as

$$v = \sqrt{\frac{e^2\rho_{sb}}{\hbar^2\Gamma}}, \qquad K = \frac{e}{\pi W\sqrt{\Gamma\rho_{sb}}}\left(\frac{1+\cos\theta_b}{4}\right)^2 = \frac{l_B}{W}\sqrt{\frac{V_0^{XY}-V_0^Z}{V_2^{XY}}}\frac{(1+\cos\theta_b)^2}{4\sin\theta_b}. \tag{S43}$$

Here, v is just the pseudospin wave velocity obtained earlier from the dispersion $\omega(k)$. The Luttinger parameter K determines the conductance $G_{cf} = Ke^2/h$. However, it is important to notice that G_{cf} describes the conductance for a counterflow/drag geometry, where opposite currents are flowing in the two edges. The helical quantum Hall exciton condensate phase does not support net transport current as long as the voltages eV are small compared to $\hbar v\pi/W$.

B. Deconfined phase

In the uncorrelated helical quantum Hall phase the transverse modes are localized within the length scale l_{dw} from the edges. Therefore, in contrast to helical quantum Hall exciton condensate phase we can define independent fields $\phi_{1(2)}(x)$ and charge densities $\rho_{1(2)}(x) = \mp\frac{e}{2\pi}\frac{\partial\phi_{1(2)}}{\partial x}$. Thus charges can be created independently on the different edges, highlighting that in the uncorrelated helical phase the charged edge excitations are deconfined.

We can now repeat the calculation done above for the confined phase. This way, assuming that $E_G(y)$ is slowly varying and that the transverse modes are described by a constant within a distance l_{dw} in the vicinity of the edge and 0 elsewhere, we arrive to Luttinger liquid Hamiltonian

$$H = \sum_{i=1}^{2}\int dx\left[\frac{e^2}{2l_{dw}\hbar^2\Gamma_{av}}\Pi_i(x)^2 + \frac{l_{dw}\rho_{s,av}}{2}(\partial_x\phi_i(x))^2\right], \tag{S44}$$

where $\rho_{s,av} = \frac{1}{l_{dw}}\int_0^{l_{dw}} dy\ V_2^{XY}\sin^2[\theta_0(y)]/\pi$ and $\Gamma_{av} = \frac{1}{l_{dw}}\int_0^{l_{dw}} dy\ e^2/\{16\pi l_B^2[(V_0^{XY} - V_0^Z)\sin^2\theta_0(y) - \frac{E_G(y)}{2}\cos\theta_0(y)]\}$. This way the Luttinger liquid parameters are identified as

$$v = \sqrt{\frac{e^2\rho_{s,av}}{\hbar^2\Gamma_{av}}}, \qquad K = \frac{e}{4\pi l_{dw}\sqrt{\Gamma_{av}\rho_{s,av}}}. \tag{S45}$$

However, in contrast to the helical quantum Hall exciton condensate phase the Luttinger liquid parameters depend strongly on the detailed shape of the $E_G(y)$ function, and if it is not slowly varying in the vicinity of the edge, there can be large corrections to the expressions above. Nevertheless, the structure of the Luttinger liquid theory (S44) is very general, and for physically reasonable parameters $K \sim 1$. Thus we expect this phase to support helical edge state transport with conductance $G \sim e^2/h$. Here G describes the conductance for a transport geometry, where a net transport current is flowing along one of the edges.

SUPPLEMENTARY NOTE 8. SPIN TEXTURE-CHARGE DENSITY RELATION IN QUANTUM HALL SYSTEMS AND NUMERICAL CALCULATION OF THE ELECTRIC CHARGE DENSITY FOR THE SPIN TEXTURES

It is known that both topological and non-topological contributions to the electric charge exist at the vortices in various systems [28–35]. It is possible to show that the mechanisms considered in these systems do not give rise to a non-topological contribution to the electric charge in quantum Hall exciton condensates. However, instead of going through the mechanisms one-by-one, we present general arguments for the topological spin texture-charge density relation and discuss the conditions for its breakdown. We complement the analytical arguments with a numerical calculation of the electric charge density for the pseudospin textures considered in the paper.

Originally the spin texture-charge density relation for quantum Hall systems was argued from the Chern-Simons relation between the density and the statistical magnetic field [36]. Namely, the electrons feel the spin texture via the additional Berry's phase and therefore the orbital degrees of freedom are influenced in the same way as if additional magnetic flux density was inserted into the system. The extra magnetic flux is associated with an extra charge yeilding to Eq. (S28) [36, 37]. This argument is valid only if the spin textures are smooth on the scale of l_B. There exist also an alternative argument for the special case where the filling factors are $\nu_\uparrow = \nu_\downarrow = 1/2$ and one neglects the higher Landau levels, so that there is a particle-hole symmetry in the system. In that case there is a general argument that a localized charge bound to a defect must be a multiple of $\pm e/2$ [38]. Therefore, the possible charges $\pm e/2$ appearing

in the special case $\nu_\uparrow = \nu_\downarrow = 1/2$ can be explained this way [39]. If the higher Landau levels are excited the particle hole symmetry is no longer exact. Therefore, this argument again relies on smoothness of the spin textures. Finally, the Eq. (S28) can be obtained with an explicit microscopic calculation [7] and it can be demonstrated for specific spin textures with the help of explicit construction of the many-particle wave functions [39]. These approaches also rely on the assumption that the spin textures are smooth on the scale of l_B.

Based on these arguments it is clear that the relation between the pseudospin texture and the charge density [Eq. (S28)] is valid if the pseudospin textures are smooth on the scale of l_B. The pseudospin textures considered in the main text [see Fig. 4 in the main text] satisfy this requirement. In fact we can explicitly construct the many-particle wave functions to illustrate how the charges $\pm\nu_\uparrow e$ appear in these textures. Namely, consider a finite system with length L and width W illustrated in Fig. 4 in the main text. Because the momentum k in the Landau level wave functions is directly connected to the position y in the real space $y = kl_B^2$, the possible values of k are $k_n = n2\pi/L$, where $n = 0, 1, ..., N$ and $N = WL/(2\pi l_B^2)$. The ground state pseudospin texture is given by $h_z(y) = h_z(kl_B^2) = \cos[\theta_0(kl_B^2)]$, $h_x(y) = h_x(kl_B^2) = \sin[\theta_0(kl_B^2)]$, $h_y(y) = 0$ so that the many particle wave-function for the ground state $|\Psi\rangle_{\rm GS}$ can be written as

$$|\Psi\rangle_{\rm GS} = \prod_{n=0}^{N} \frac{1}{\sqrt{2\{1-\cos[\theta_0(k_n l_B^2)]\}}} \Big\{ \sin[\theta_0(k_n l_B^2)]\hat{\psi}^\dagger_{k_n,\uparrow} + \big[1-\cos[\theta_0(k_n l_B^2)]\big]\hat{\psi}^\dagger_{k_n,\downarrow}\Big\}|0\rangle. \tag{S46}$$

The elementary excitation is described by a pseudospin texture $h_z(y) = h_z(kl_B^2) = \cos[\theta_0(kl_B^2)]$, $h_x(x,y) = h_x(x,kl_B^2) = \sin[\theta_0(kl_B^2)]\cos[\phi(x)]$, $h_y(x,y) = h_y(x,kl_B^2) = \sin[\theta_0(kl_B^2)]\sin[\phi(x)]$, where $\phi(x) = 2\pi x/L$. The winding of $\phi(x)$ can be removed by introducing a momentum shift $\Delta k = 2\pi/L$ between the electron and hole Landau level wave functions. Because the pseudospin-texture is slowly varying and the pseudopin close to the edge points down the many-particle wave function for the excited state can be written as

$$|\Psi\rangle_{\rm ES} = \hat{\psi}^\dagger_{k_0,\downarrow} \prod_{n=0}^{N-1} \frac{1}{\sqrt{2\{1-\cos[\theta_0(k_n l_B^2)]\}}} \Big\{ \sin[\theta_0(k_n l_B^2)]\hat{\psi}^\dagger_{k_n,\uparrow} + \big[1-\cos[\theta_0(k_n l_B^2)]\big]\hat{\psi}^\dagger_{k_{n+1},\downarrow}\Big\}|0\rangle. \tag{S47}$$

From these expression one finds that the charge density is

$$\delta\rho(y) = -\frac{e}{4\pi l_B^2}\Big\{\cos\big[\theta_0(y+\frac{2\pi}{L}l_B^2)\big] - \cos\big[\theta_0(y)\big]\Big\} = -\frac{e}{4\pi}\frac{\partial\phi}{\partial x}\frac{\partial\cos\big[\theta_0(y)\big]}{\partial y} \tag{S48}$$

in agreeement with Eq. (S29). From this expression it straightforwardly follows that the charges appearing at each edge for the pseudospin texture shown in Fig. 4 in the main text are $\pm\nu_\uparrow e$, where $\nu_\uparrow$ is the bulk filling factor for pseudospin up. It is also clear that in a closed system the charges of the possible edge excitations and the charges of the vortices must be related. As shown in Fig. 5 in the main text it is possible to end the charged edge excitation into a bulk vortex, and the total charge in the system must be an integer multiple of e. This requirement is satisfied because the possible charges of the vortices are $\pm\nu_{\uparrow(\downarrow)}e$ and $\nu_\uparrow + \nu_\downarrow = 1$.

In all arguments so far the whole pseudospin texture was considered to be smooth on the scale of l_B. However, it turns out that it is possible to relax this assumption. Namely, if one considers the type of pseudospin textures shown in Fig. 4 in the main text it is actually enough that *the pseudospin texture is smooth on the scale of l_B in the bulk region between the localized charges.* Close to the edge it may vary arbitrarily sharply. To understand this consider first the type of smooth pseudospin texture shown in Fig. 4, such that charge $\nu_\uparrow e$ appears at one edge and $-\nu_\uparrow e$ at the other. We can now deform one end of the pseudospin texture in such a way that the rest of the pseudospin texture remains smooth. Then, there is still charge $\nu_\uparrow e$ localized on one edge and the bulk is charge neutral, which means that a charge $-\nu_\uparrow e$ necessarily remains at the other edge although it is no longer smooth on the scale of l_B. The values of the polarization charges in this system are therefore fully topological. No local perturbation in the pseudospin texture can change the charges appearing at the edges. Only a global perturbation which connects the fractionally charged excitations may give rise to redistribution of the charges and modify their values from $\pm\nu_\uparrow e$.

We have numerically verified the statements based on the analytical arguments given above. For this purpose we have inserted an order parameter term

$$H_\Delta = -\Delta_0\Big[h_z(x,y)\sigma_z\tau_0 + h_x(x,y)\sigma_x\tau_x + h_y(x,y)\sigma_x\tau_y\Big] \tag{S49}$$

to the BHZ Hamiltonian [Eq. (S1)]. The charge density associated with the excitation $\delta\rho(\mathbf{r})$ can be numerically computed by calcuting the difference between the charge densities for pseudospin textures $h_z(x,y) = \cos[\theta(y)]$, $h_x(x,y) = \sin[\theta(y)]\cos[\phi(x)]$, $h_y(x,y) = \sin[\theta(y)]\sin[\phi(x)]$, where $\phi(x) = 2\pi x/l_\phi$ (excited state) and $\phi(x) = 0$ (ground

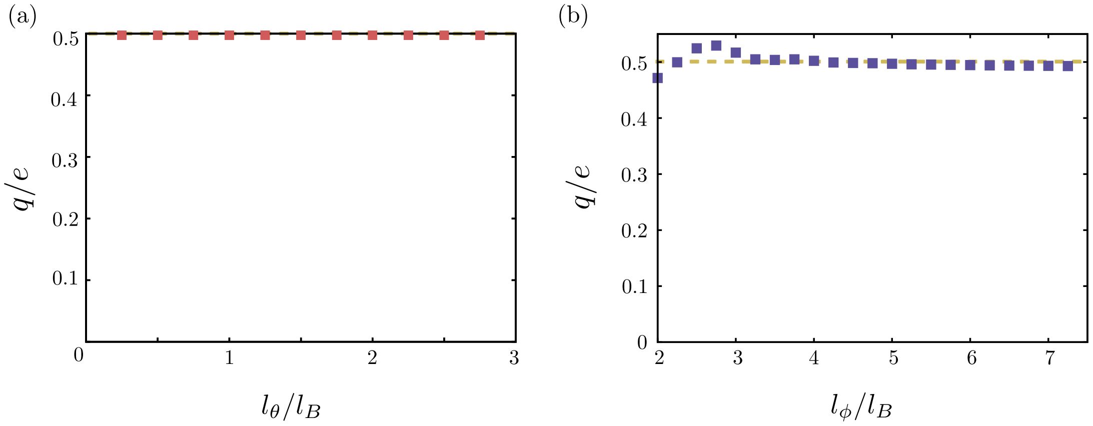

Supplementary Fig. 2. **Charge of the edge excitation in a closed system.** Charge concentrated close to the edge of the sample as a function of (a) l_θ and (b) l_ϕ. Here l_θ determines the length scale where the angle $\theta(y)$ is varied from π to θ_b in the vicinity of the edge and l_ϕ describes length scale where the angle $\phi(x)$ rotates by 2π. The charge q is computed by integrating the charge density over a single period l_ϕ along the x-direction and over the interval of width $5l_B$ from the edge of the sample in y-direction. We have chosen $\theta_b = \pi/2$ so that the expected charge shown by a dotted line is $q = \nu_\uparrow e = e/2$. As expected based on the analytical arguments the value of q is independent on l_θ (as long as the width of the integration interval in y-direction is chosen to be larger than the width of the domain wall where the charge is concentrated in the vicinity of the edge), and deviations from the value $e/2$ as a function of l_ϕ start to occur only when $l_\phi \lesssim 4l_B$. In the numerics we have chosen the order parameter strength $\Delta_0 = 1$ meV, the width of the sample $W = 20l_B$, and the space is discretized using a lattice constant $0.25l_B$. For (a) we fix $l_\phi = 5l_B$ and for (b) $l_\theta = 3l_B$. The parameters of the BHZ Hamiltonian are same as in Supplementary Fig. 1.

state). Here we have introduced a length scale l_ϕ where the phase $\phi(x)$ rotates over 2π. The charge of an elementary excitation q is obtained by integrating the charge density over a period l_ϕ in x-direction and over the width of the domain wall in y-direction. If we choose $l_\phi \gg l_B$ the charge of the excitation according to our analytic arguments should be $q = \pm\nu_\uparrow e$. However, l_ϕ allows us also to control how fast the pseudospin direction changes in such a way that it influences the system everywhere in the bulk. Therefore, if we choose $l_\phi \lesssim l_B$ it is possible to excite the higher Landau levels everywhere in the bulk so that topological protection is destroyed, and we can study how this affects the charge of the excitation. Additionally we can illustrate the topological nature of the charge of the pseudospin texture by demonstrating that deformations of the pseudospin texture appearing only close to the edge do not affect the value of q. For this purpose we define

$$\theta(y) = \begin{cases} \pi + (\theta_b - \pi)y/l_\theta, & 0 < y < l_\theta \\ \theta_b, & l_\theta < y < W - l_\theta \\ \theta_b + (\pi - \theta_b)(y - W + l_\theta)/l_\theta, & W - l_\theta < y < W \end{cases} \tag{S50}$$

in such a way that l_θ allows to control the length scale where the angle $\theta(y)$ is varied from π to θ_b in the vicinity of the edge. Based on our topological arguments the charge q should be independent of l_θ. (We assume $W \gg l_\theta$.)

The numerical results are shown in Supplementary Figs. 2. As can be seen from these figures for smooth pseudospin textures the numerical results are in agreement with the analytical expectations. Moreover, q does not depend on l_θ [Supplementary Fig. 2(a)] in agreement with the topological argument. On the other hand, if we deform the pseudospin texture in such a way that it rotates fast in the bulk by choosing $l_\phi \lesssim 4l_B$ it is possible to excite the higher Landau levels everywhere in the bulk so that topological protection is destroyed. This results in redistribution of the charges giving rise to deviations of q from the value $\pm\nu_\uparrow e$ [Supplementary Fig. 2(b)].

[1] B. A. Bernevig, T. L. Hughes, and S.-C. Zhang, *"Quantum Spin Hall Effect and Topological Phase Transition in HgTe Quantum Wells"*, Science **314**, 1757-1761 (2006).

[2] C. Liu, T. L. Hughes, X.-L. Qi, K. Wang, and S.-C. Zhang, *"Quantum Spin Hall Effect in Inverted Type-II Semiconductors"*, Phys. Rev. Lett. **100**, 236601 (2008).

[3] L. Du, I. Knez, G. Sullivan, and R.-R. Du, Phys. Rev. Lett. **114**, *"Robust Helical Edge Transport in Gated InAs/GaSb Bilayers"*, 096802 (2015).

[4] E. M. Spanton, K. C. Nowack, L. Du, G. Sullivan, R.- R. Du, and K. A. Moler, *"Images of Edge Current in InAs/GaSb Quantum Wells"*, Phys. Rev. Lett. **113**, 026804 (2014).

[5] P. Michetti, J. C. Budich, E. G. Novik, and P. Recher, *"Tunable quantum spin Hall effect in double quantum wells"*, Phys. Rev. B **85**, 125309 (2012).

[6] C. Liu and S.-C. Zhang, in *Topological Insulators*, edited by M. Franz and L. W. Molenkamp (Elsevier, Amsterdam, 2013).

[7] K. Moon et al., *"Spontaneous interlayer coherence in double-layer quantum Hall systems: Charged vortices and Kosterlitz-Thouless phase transitions"*, Phys. Rev. B **51**, 5138 (1995).

[8] I. B. Spielman, L. A. Tracy, J. P. Eisenstein, L. N. Pfeiffer, and K. W. West, *"Spin Transition in Strongly Correlated Bilayer Two-Dimensional Electron Systems"*, Phys. Rev. Lett. **94**, 076803 (2005).

[9] P. Giudici, K. Muraki, N. Kumada, Y. Hirayama, and T. Fujisawa, *"Spin-Dependent Phase Diagram of the $\nu_T = 1$ Bilayer Electron System"*, Phys. Rev. Lett. **100**, 106803 (2008).

[10] A. D. K. Finck, J. P. Eisenstein, L. N. Pfeiffer, and K. W. West, *"Quantum Hall Exciton Condensation at Full Spin Polarization"*, Phys. Rev. Lett. **104**, 016801 (2010).

[11] L. Tiemann, W. Wegscheider, and M. Hauser, *"Electron Spin Polarization by Isospin Ordering in Correlated Two-Layer Quantum Hall Systems"*, Phys. Rev. Lett. **114**, 176804 (2015).

[12] F. Qu *et al.*, *"Electric and Magnetic Tuning Between the Trivial and Topological Phases in InAs/GaSb Double Quantum Wells"*, Phys. Rev. Lett. **115**, 036803 (2015).

[13] L. Du, W. Lou, K. Chang, G. Sullivan, R-R. Du, *"Gate-Tuned Spontaneous Exciton Insulator in Double-Quantum Wells"*, arXiv:1508.04509 (2015).

[14] F. Nichele *et al.*, *"Insulating State and Giant Nonlocal Response in an InAs/GaSb Quantum Well in the Quantum Hall Regime"*, Phys. Rev. Lett. **112**, 036802 (2014).

[15] S. Q. Murphy, J. P. Eisenstein, G. S. Boebinger, L. N. Pfeiffer, and K. W. West, *"Many-body integer quantum Hall effect: Evidence for new phase transitions"*, Phys. Rev. Lett. **72**, 728 (1994).

[16] I. B. Spielman, J. P. Eisenstein, L. N. Pfeiffer, and K. W. West, *"Resonantly Enhanced Tunneling in a Double Layer Quantum Hall Ferromagnet"*, Phys. Rev. Lett. **84**, 5808 (2000).

[17] M. Kellogg, I. B. Spielman, J. P. Eisenstein, L. N. Pfeiffer, and K. W. West, *"Observation of Quantized Hall Drag in a Strongly Correlated Bilayer Electron System"*, Phys. Rev. Lett. **88**, 126804 (2002).

[18] A. R. Champagne, J. P. Eisenstein, L. N. Pfeiffer, and K. W. West, *"Evidence for a Finite-Temperature Phase Transition in a Bilayer Quantum Hall System"*, Phys. Rev. Lett. **100**, 096801 (2008).

[19] L. Tiemann, Y. Yoon, W. Dietsche, K. von Klitzing, and W. Wegscheider, *"Dominant parameters for the critical tunneling current in bilayer exciton condensates"*, Phys. Rev. B **80**, 165120 (2009).

[20] P. R. Eastham, N. R. Cooper, and D. K. K. Lee, *"Vortex states of a disordered quantum Hall bilayer"*, Phys. Rev. B **80**, 045302 (2009).

[21] J. Sun, G. Murthy, H. A. Fertig, and N. Bray-Ali, *"Bilayer quantum Hall ferromagnet in a periodic potential"*, Phys. Rev. B **81**, 195314 (2010).

[22] A. Stern, S. M. Girvin, A. H. MacDonald, and N. Ma, *"Theory of Interlayer Tunneling in Bilayer Quantum Hall Ferromagnets"*, Phys. Rev. Lett. **86**, 1829 (2001).

[23] H. A. Fertig and G. Murthy, *"Coherence Network in the Quantum Hall Bilayer"*, Phys. Rev. Lett. **95**, 156802 (2005).

[24] P. R. Eastham, N. R. Cooper, and D. K. K. Lee, *"Critical Supercurrents and Self-Organization in Quantum Hall Bilayers"*, Phys. Rev. Lett. **105**, 236805 (2010).

[25] T. Hyart and B. Rosenow, *"Quantitative description of Josephson-like tunneling in $\nu_T = 1$ quantum Hall bilayers"*, Phys. Rev. B **83**, 155315 (2011).

[26] T. Hyart and B. Rosenow, *"Influence of Topological Excitations on Shapiro Steps and Microwave Dynamical Conductance in Bilayer Exciton Condensates"*, Phys. Rev. Lett. **110**, 076806 (2013).

[27] T. Giamarchi, *Quantum Physics in One Dimension*, (Oxford University Press, 2003).

[28] D.I. Khomskii and A. Freimuth, *"Charged Vortices in High Temperature Superconductors"*, Phys. Rev. Lett. **75**, 1384 (1995).

[29] G. Blatter, M. Feigelman, V. Geshkenbein, A. Larkin, and A. van Otterlo, *"Electrostatics of Vortices in Type-II Superconductors"*, Phys. Rev. Lett. **77**, 566 (1996).

[30] K. Kumagai, K. Nozaki, and Y. Matsuda, *"Charged vortices in high-temperature superconductors probed by NMR"*, Phys. Rev. B **63**, 144502 (2001).

[31] V. D. Natsik, *"Electric activity of vortices in superfluid ^4He"*, Low Temp. Phys. **31**, 915 (2005).

[32] S. I. Shevchenko, A. S. Rukin, *"On the electric activity of superfluid systems"*, JETP Lett. **90**, 42-46 (2009).

[33] G.E. Volovik, *"Spontaneous electrical polarization of vortices in superfluid ^3He"*, JETP Lett. **39**, 200-203 (1984).

[34] A. S. Rukin, S. I. Shevchenko, *"On electric fields created by quantized vortices"*, Low Temp. Phys. **37**, 884-888 (2011),

[35] I. N. Adamenko and E. K. Nemchenko, *"Electric polarization of He II by quantized vortices"*, Low Temp. Phys. **41**, 495-501 (2015).

[36] S. Sondhi, A. Karlhede, S. Kivelson, and E. Rezayi, *"Skyrmions and the crossover from the integer to fractional quantum Hall effect at small Zeeman energies"*, Phys. Rev. B **47**, 16419 (1993).

[37] S. Girvin, The Quantum Hall Effect: Novel Excitations and Broken Symmetries, cond-mat/9907002. IUCM-98- 010 (Indiana Univ., Bloomington, IN, 1999).

[38] C.-Y. Hou, C. Chamon, and C. Mudry, *"Electron Fractionalization in Two-Dimensional Graphenelike Structures"*, Phys. Rev. Lett. **98**, 186809 (2007).

[39] S. M. Girvin and A. H. MacDonald, Perspectives in Quantum Hall Effects, edited by S. Das Sarma and A. Pinczuk (Wiley,

New York, 1997), Chap. V; arXiv:cond-mat/9505087.
